# Gene variations in oestrogen pathways, *CYP19A1*, daily 17β-estradiol and mammographic density phenotypes in premenopausal women

**DOI:** 10.1186/s13058-014-0499-2

**Published:** 2014-12-19

**Authors:** Vidar G Flote, Anne-Sofie Furberg, Anne McTiernan, Hanne Frydenberg, Giske Ursin, Anita Iversen, Trygve Lofteroed, Peter T Ellison, Erik A Wist, Thore Egeland, Tom Wilsgaard, Karen W Makar, Jenny Chang-Claude, Inger Thune

**Affiliations:** 1 The Cancer Centre, Oslo University Hospital, Oslo, N-0424 Norway; 2 Department of Community Medicine, Faculty of Health Sciences, UiT, The Arctic University of Norway, Tromsø, N-9037 Norway; 3 Fred Hutchinson Cancer Research Center, Public Health Sciences Division, Seattle, 98109-1024 WA USA; 4 Cancer Registry of Norway, Majorstuen, Oslo N-0304 Norway; 5 Faculty of Health Sciences, UiT, The Arctic University of Norway, Tromsø, N-9037 Norway; 6 Department of Anthropology, Harvard University, Cambridge, 02138 MA USA; 7 Department of Chemistry, Norwegian University of Life Sciences, Biotechnology and Food Science, Aas, N-1432 Norway; 8 Unit of Genetic Epidemiology, Division of Cancer Epidemiology, Deutches Krebsforschungszentrum, Heidelberg, 69120 Germany

## Abstract

**Introduction:**

High mammographic density is an established breast cancer risk factor, and circulating oestrogen influences oestrogen-regulating gene expression in breast cancer development. However, less is known about the interrelationships of common variants in the *CYP19A1* gene, daily levels of oestrogens, mammographic density phenotypes and body mass index (BMI) in premenopausal women.

**Methods:**

Based on plausible biological mechanisms related to the oestrogen pathway, we investigated the association of single nucleotide polymorphisms (SNPs) in *CYP19A1*, 17β-estradiol and mammographic density in 202 premenopausal women. DNA was genotyped using the Illumina Golden Gate platform. Daily salivary 17β-estradiol concentrations were measured throughout an entire menstrual cycle. Mammographic density phenotypes were assessed using a computer-assisted method (Madena). We determined associations using multivariable linear and logistic regression models.

**Results:**

The minor alleles of *rs749292* were positively (*P* = 0.026), and the minor alleles of *rs7172156* were inversely (*P* = 0.002) associated with daily 17β-estradiol. We observed an 87% lower level of daily 17β-estradiol throughout a menstrual cycle in heavier women (BMI >23.6 kg/m^2^) of *rs7172156* with minor genotype *aa* compared with major genotype *AA*. Furthermore, the *rs749292* minor alleles were inversely associated with absolute mammographic density (*P* = 0.032). Lean women with *rs749292* minor alleles had 70 to 80% lower risk for high absolute mammographic density (>32.4 cm^2^); *Aa*: odds ratio (OR) = 0.23 (95% CI 0.07 to 0.75). Lean women with *rs7172156* minor homozygous genotype had OR 5.45 for high absolute mammographic density (*aa*: OR = 5.45 (95% CI 1.13 to 26.3)).

**Conclusion:**

Our findings suggest that two SNPs in *CYP19A1*, *rs749292* and *rs7172156*, are associated with both daily oestrogen levels and mammographic density phenotypes. BMI may modify these associations, but larger studies are needed.

**Electronic supplementary material:**

The online version of this article (doi:10.1186/s13058-014-0499-2) contains supplementary material, which is available to authorized users.

## Introduction

Sex hormones, in particular oestrogens, are associated with breast cancer development in both pre- and postmenopausal women [1]-[3], and circulating oestrogens have been shown to influence oestrogen-regulating gene expression [4]. *CYP19A1* is a member of the cytochrome P450 family and is involved in the bioconversion of androstenedione to oestrone and of testosterone to estradiol [5]. Human *CYP19A1* is a protein commonly known as aromatase and is a gene product of *CYP19A1*, which is located on chromosome 15q21.2 [6]. In humans, aromatase is expressed in the gonads, adipose tissue and other sites, although the primary site of oestrogen production in premenopausal women is the ovaries [5]. Breast adipose tissue produces oestrogen locally, which may be increased in pre- and postmenopausal obese women [7], owing to higher levels of proinflammatory cytokines such as tumour necrosis factor, a known inducer of aromatase [8],[9]. Importantly, the gene *CYP19A1* is polymorphic, and the presence of single-nucleotide polymorphisms (SNPs) in the gene may alter aromatase activity, thereby causing variations in the levels of oestrogens [10].

Endogenous oestrogen has been shown to be both inversely [11],[12], and positively [13]-[15] associated with mammographic density, and both high absolute and percent mammographic density have consistently been associated with breast cancer development [16],[17]. Furthermore, mammographic density phenotypes are a strong heritable biomarker of breast cancer development, and twins studies suggest that genetic factors account for 30% to 60% of its variance [18],[19]. In a recent meta-analysis including five genome-wide association studies, a variant (*rs10995190*) in the *ZNF365* gene, which promotes genome stability during DNA damage, was associated with both breast cancer risk and mammographic density [20]. However, this SNP explains only 0.5% of the variance of mammographic density, and many other loci may be involved in predicting mammographic density phenotypes and breast cancer development [20].

Mammographic density is also influenced by several well-known major breast cancer risk factors such as age, body mass index (BMI), parity, and hormone therapy [21]. Previous studies have observed an inverse association between BMI and premenopausal breast cancer development [22],[23]. In contrast, weight gain in early adult life has been associated with postmenopausal breast cancer development [24], but the association between weight gain and premenopausal breast cancer development has not yet been clarified [25]. However, premenopausal abdominal adiposity has been associated with oestrogen receptor–negative (ER−) breast cancer [26]. Studies also support excess weight being associated with higher oestrogen levels and ER+ postmenopausal breast cancer development [27],[28]. In addition, we have previously shown that salivary estradiol concentrations are positively associated with BMI throughout the menstrual cycle in premenopausal women [29].

Few studies have been focused on genetic susceptibility, daily levels of oestrogen and premenopausal mammographic density, but plausible biological mechanisms may exist because functional genetic polymorphisms in the aromatase gene *CYP19A1* have been associated with higher estradiol levels. Therefore, the main aim of the present study was to elaborate whether hypothesis-driven selected common variants in the *CYP19A1* gene are associated with daily 17β-estradiol levels and mammographic density phenotypes among healthy premenopausal women and whether BMI modifies these associations.

## Methods

A total of 204 women ranging in age from 25 to 35 years participated in the Norwegian Energy Balance and Breast cancer Aspects I study (EBBA-I) from 2000 to 2002 at the Department of Clinical Research, University Hospital of North Norway, Tromsø (UNN) [30]. Women meeting the following eligibility criteria were included: self-reported regular menstruation (normal cycle length, 22 to 38 days within the previous 3 months), no ongoing use of steroid contraceptives, no pregnancy or lactation in the previous 6 months, no history of gynaecological disorders and no chronic disorders (for example, diabetes, hypo- or hyperthyroidism). Two women were excluded because of missing mammographic data, resulting in 202 participants being included in the present study.

Participants’ characteristics, including reproductive and lifestyle factors, were collected by one trained nurse using questionnaires and interviews at the time of recruitment. Recall and memory-probing aids, including a lifetime calendar, were used to date specific life events. Questionnaires (filled out by the participant and interviewer, administered by trained personnel) were used to collect information about birth weight, age at menarche, marital status, education, ethnicity, reproductive history, lifetime total physical activity, previous use of hormonal contraceptives and family history of cancer, smoking and alcohol. Dietary data were collected on 7 different days during the menstrual cycle (days 3 to 6 and 21 to 23) using a previously validated, precoded food diary [31]. Daily average energy and nutrient intake were computed.

### Clinical parameters

Participants attended three study visits during one menstrual cycle: first visit, days 1 to 5 of the menstrual cycle, early follicular phase; second visit, days 7 to 12, late follicular phase; and third visit, days 21 to 25, late luteal phase. Measurements included height to the nearest 0.5 cm and weight (in light clothing) to the nearest 0.1 kg on a regularly calibrated electronic scale. BMI in kilograms per square metre was calculated for all participants. Fasting blood samples were drawn at all three scheduled visits during the menstrual cycle.

### Assessment of oestrogen

Serum concentrations of 17β-estradiol were measured in fresh sera for all three collection points using a direct immunometric assay (Immuno-1; Bayer Diagnostics, Norway) at the Department of Clinical Chemistry, UNN [30]. The sensitivity was 0.01 nmol/L, and the coefficient of variation (CV) was 3.9%.

To assess the bioavailable fraction of 17β-estradiol, the participants collected daily saliva samples during one menstrual cycle, preferably in the morning, starting on the first day of menstrual bleeding according to previously established and validated collection protocols developed at the Reproductive Ecology Laboratory of Harvard University [32] and according to the manufacturer’s protocol [30]. The samples were stored at −70°C. All samples were run in duplicate, and samples from the same cycles were run within the same assay. The assays were done in different batches at Harvard University. 17β-estradiol concentrations were measured in daily saliva samples using a ^125^I-based radioimmunoassay kit (no. 39100; Diagnostic Systems Laboratory, Webster, TX, USA). All cycles were aligned to the day of ovulation, based on the identification of the 17β-estradiol drop, which provides a reasonable estimate of the day of ovulation [33],[34]. The midcycle 17β-estradiol drop could not be made for 14 of the included women, and their cycles were not aligned. Overall mean salivary 17β-estradiol concentration was calculated for all participants, whereas an additional index of mean menstrual estradiol on days −7 to +6 was calculated for the 188 women with aligned cycles. The sensitivity of the 17β-estradiol salivary assay was 4 pmol/L, and the average intra-assay CV was 9%. The measurements of 17β-estradiol had a higher CV at the start and end of the menstrual cycle, and the interassay CV ranged from 23% (low pool) to 13% (high pool). Furthermore, there were higher rates of missing data at the end of the cycle, so we included aligned measurements of salivary 17β-estradiol from day −7 to day +6 in this study.

### Assessment of mammographic density

Bilateral two-view mammograms were obtained from women during the second scheduled visit (between cycle days 7 and 12) at the Centre of Breast Imaging, UNN, using a standard protocol [30]. The left craniocaudal mammograms were digitised and imported into a computerised mammographic density assessment programme (Madena) developed at the University of Southern California School of Medicine (Los Angeles, CA, USA) [35],[36]. The density measurements were conducted by one trained reader (GU), and the total breast area was determined by a research assistant trained by GU. The total breast area was defined using a special outlining tool, and the size of this area in square centimetres using the Madena software. To assess density, the reader outlined a region of interest (ROI), excluding the pectoralis muscle, prominent veins and fibrous strands. The reader applied a tinting tool to pixels considered to represent dense areas of the mammograms within the ROI. The Madena software calculated the size of this dense area in square centimetres. Absolute mammographic breast density represented this dense area, and the percentage mammographic density was the ratio of absolute mammographic breast density to total breast area multiplied by 100. The mammograms were read in four batches, with an equal number of mammograms included in each batch. A duplicate reading of 26 randomly selected mammograms from two of the batches showed a Pearson’s correlation coefficient of 0.97. The reader was blinded to any characteristics of the study population.

### Single-nucleotide polymorphism selection and genotyping

We analysed *CYP19A1* genetic polymorphisms that encode the aromatase enzyme. Blood samples from 204 women in the EBBA-I study were frozen at −70°C. DNA was extracted from whole blood using a MagAttract DNA Blood Mini M48 kit (QIAGEN, Valencia, CA, USA) by the Department of Medical Genetics, UNN. DNA was genotyped on the Golden Gate Platform (Illumina, San Diego, CA, USA) at the Fred Hutchinson Cancer Research Center (Makar Lab), using the manufacturer’s protocol. In brief, 250 ng of genomic DNA was divided into aliquots in 96-well plates, processed accordingly and scanned on the Illumina iScan reader using BeadStudio software.

We conducted a series of quality control procedures [37]. SNP call rates exceeded 99% for this study, with 100% concordance of blinded duplicates. The linkage disequilibrium select algorithm was employed to choose the tag SNPs via the Genome Variation Server [38],[39]. The SNPs were selected using an *r* ^2^ threshold of 0.8 and a minor allele frequency >5%, representing variability in the white European population. Tag SNP coverage extended 2 kilobases (kb) upstream and 1 kb downstream of the gene, and 29 SNPs were covered. We further reduced the number of SNPs using power calculations and ended up with a final selection of eight common SNPs with minor allele frequency >0.2: *rs10046*, *rs17703883*, *rs2414097*, *rs2445761*, *rs4646*, *rs7172156*, *rs727479* and *rs749292* (see Additional file 1). None of the selected SNPs was monomorphic or significantly out of Hardy–Weinberg equilibrium.

### Covariate analytes

Serum concentrations of total cholesterol were determined enzymatically using cholesterol esterase and cholesterol oxidase. High-density lipoprotein cholesterol (HDL-C) was quantified by direct assay using enzymes modified by polyethylene glycol and dextran sulphate.

### Statistical methods

On the basis of the plausible biological mechanisms related to the oestrogen pathway, we investigated the associations between eight SNPs in the *CYP19A1* gene, hormone levels (salivary midmenstrual 17β-estradiol and serum 17β-estradiol) and mammographic density phenotypes (total breast area, absolute mammographic density, percent mammographic density and nondense breast area) using multivariable linear regression models. Associations were assessed for the selected SNPs, and the selected SNPs were coded as *AA* = 0 (major homozygous), *Aa* = 1 (heterozygous) and *aa* = 2 (minor homozygous) and were included as ordinal variables in the models. We compared the linear response between the categories of genotypes by including indicator variables for *Aa* and *aa*, using *AA* as the reference.

Age, parity and BMI are known to be associated with mammographic density phenotypes, are possibly associated with hormone levels and/or CYP19A1 variants, and were therefore considered as potential confounders and included as covariates in all models [21]. Furthermore, the models with mammographic density as the dependent variable also included salivary 17β-estradiol and serum HDL-C, both of which are known to influence mammographic density [40],[13]. In the final analyses, we focused on two selected SNPs (*rs7172156* and *rs749292*) and stratified the women by major, heterozygous and minor genotypes. We then compared the genotype groups using different characteristics of the study population (lifestyle factors, anthropometric measures, serum blood sampling and salivary hormone sampling), and we used one-way analysis of variance for continuous variables and the χ^2^ test for categorical variables.

The multivariable logistic regression models were run using median absolute mammographic density (32.4 cm^2^) and median percent mammographic density (28.5%) as cutoff values. Mammographic density was used as a dependent variable, and *rs7172156* and *rs749292* were used as independent variables, adjusted for age, parity and BMI. In addition, we analysed in detail whether BMI variations influenced our results (that is, tertiles/dichotomised BMI), but only dichotomised BMI by median BMI values gave additional information and thus were included in the final analysis.

We used linear mixed models for repeated measures to study variations of daily salivary 17β-estradiol across the menstrual cycle for subgroups of women with major, minor homozygous or heterozygous genotypes in the SNPs *rs7172156* and *rs749292*, and we then adjusted for age, BMI and parity. The Toeplitz covariance structure gave the best fit to the data and was used in all models.

Our candidate polymorphisms were based on plausible biological hypotheses, and all *P*-values were two-tailed and considered significant when the value was <0.05. The analyses were conducted with SPSS version 21.0 software (IBM, Armonk, NY, USA).

### Ethical considerations

All participants underwent informed consent procedures and signed a consent form. The study was approved by the Norwegian Data Inspectorate and the Regional Committee for Medical Research Ethics.

## Results

The participating premenopausal women had mean values (standard deviation (SD)) for age of 30.7 (3.07) years and BMI of 24.4 (3.77) kg/m^2^ (Table 1). When we stratified the women into groups for *rs749292* and *rs7172156* by major homozygous, heterozygous and minor homozygous genotypes, we observed no differences in lifestyle factors, anthropometric measures or serum analytes (Table 1).

**Table 1:** **Characteristics of the Norwegian EBBA-I study population overall and by** ***CYP19A1*** **single-nucleotide polymorphisms** ***rs7172156*** **and** ***rs749292***

Study characteristics		Overall means (SD)	***rs7172156***				***rs749292***			
			Major genotype, ***AA***	Heterozygous genotype, ***Aa***	Minor genotype, ***aa***	***P*** -value ^b^	Major genotype, ***AA***	Heterozygous genotype, ***Aa***	Minor genotype, ***aa***	***P*** -value ^b^
			( ***n*** = 82) ^a^	( ***n*** = 91) ^a^	( ***n*** = 31) ^a^		( ***n*** = 62 ) ^a^	( ***n*** = 93) ^a^	( ***n*** = 48) ^a^	
Age (yr)		30.7 (3.07)	30.2 (3.09)	31.1 (3.12)	30.7 (2.79)	0.149	30.5 (2.99)	31.0 (3.17)	30.4 (3.00)	0.425
Education (total yr)		16.1 (3.01)	15.9 (2.65)	16.2 (3.41)	16.3 (2.73)	0.701	15.8 (3.02)	16.3 (3.15)	16.2 (2.70)	0.603
Reproductive factors^c^										
Age at menarche (yr)		13.1 (1.36)	13.1 (1.40)	13.2 (1.43)	13.1 (1.04)	0.793	13.0 (1.14)	13.2 (1.52)	13.2 (1.20)	0.536
Menstrual cycle length (days)		28.3 (3.42)	28.7 (3.01)	28.2 (3.66)	27.8 (3.69)	0.463	28.0 (3.48)	28.3 (3.50)	28.8 (3.22)	0.503
Number of children		0.91 (1.13)	0.85 (1.17)	0.98 (1.11)	0.84 (1.10)	0.721	0.73 (1.01)	0.99 (1.12)	0.98 (1.28)	0.320
Weight at birth (g)		3,389 (561)	3,428 (554)	3,369 (585)	3,343 (519)	0.701	3,274 (574)	3,507 (530)	3,328 (556)	0.024
Clinical parameters										
BMI (kg/m^2^)^d^		24.4 (3.77)	24.4 (3.74)	24.2 (3.73)	25.0 (4.00)	0.606	24.8 (4.66)	24.1 (3.19)	24.3 (3.33)	0.467
Total tissue fat (%) (DXA)^e^		34.2 (7.62)	33.9 (7.69)	33.7 (7.92	36.0 (6.41)	0.328	35.1 (8.10)	33.5 (7.51)	33.9 (7.08)	0.455
Serum samples^f^										
Total cholesterol (mmol/L)		4.45 (0.78)	4.55 (0.84)	4.36 (0.75)	4.40 (0.71)	0.268	4.45 (0.77)	4.33 (0.79)	4.68 (0.76)	0.044
HDL-C (mmol/L)		1.54 (0.33)	1.55 (0.30)	1.54 (0.36)	1.51 (0.34)	0.833	1.53 (0.32)	1.54 (0.36)	1.55 (0.31)	0.940
Serum hormones^f^										
Estradiol (nmol/L)		0.15 (0.06)	0.15 (0.06)	0.15 (0.07)	0.14 (0.06)	0.644	0.14 (0.06)	0.15 (0.07)	0.14 (0.06)	0.646
SHBG (nmol/L)		51.9 (19.5)	51.7 (18.1)	52.7 (22.0)	50.2 (15.3)	0.828	51.6 (17.0)	53.3 (22.8)	50.0 (15.5)	0.626
**Salivary hormones** ^**g**^										
Midmenstrual estradiol (pmol/L)		18.2 (8.98)	19.4 (9.52)	19.0 (8.81)	12.6 (5.39)	0.001	16.3 (7.67)	18.4 (9.59)	19.8 (9.03)	0.095
**Lifestyle factors** ^**c**^										
Current smokers (%)		22.3	19.3	22.8	28.1	0.586	13.8	23.2	10.8	0.768
Alcohol (U/wk)		2.89 (3.38)	3.03 (3.41)	2.84 (3.38)	2.67 (3.38)	0.865	2.52 (3.07)	3.07 (3.41)	3.08 (3.74)	0.561
Energy intake (kJ/day)		8,093 (1,900)	8,371 (1,837)	8,085 (1,754)	7,381 (2,314)	0.046	7,749 (1,975)	8,087 (2,005)	8,495 (1,480)	0.123
Previous use of OC (%)		83.4	81.9	85.7	81.2	0.747	81.0	83.9	85.7	0.788
Leisure time MET (hr/wk)		57.6 (88.6)	68.2 (133)	48.4 (32.0)	56.7 (42.9)	0.337	51.9 (39.4)	63.4 (125)	53.6 (36.8)	0.685
**Mammographic density** ^**e**^										
Total area (cm^2^)		137 (62.5)	131 (64.9)	137 (59.6)	155 (62.8)	0.209	149 (69.5)	132 (61.1)	129 (52.6)	0.161
Absolute density (cm^2^)		34.7 (23.4)	34.7 (22.4)	32.8 (23.8)	40.7 (24.4)	0.283	39.1 (26.2)	33.5 (23.8)	32.3 (17.4)	0.238
Percent density (%)		29.8 (19.0)	31.5 (19.0)	28.6 (20.4)	28.8 (14.5)	0.594	30.1 (18.1)	29.8 (20.2)	29.9 (17.9)	0.995

Numbers in parentheses are standard deviations (SDs). BMI, body mass index; E_2_, 17β-estradiol; DXA, Dual-energy X-ray absorptiometry; HDL-C, High-density lipoprotein-cholesterol; LDL-C, Low-density lipoprotein-cholesterol; MET, Metabolic equivalent; OC, Oral contraceptives; SHBG, Sex hormone-binding globulin. ^a^Numbers may vary due to missing information. ^b^OneWay ANOVA or χ^2^ test, significance level *P* < 0.05. ^c^Questionnaires. ^d^Measurements at days 1 to 5 after onset of menstrual cycle. ^e^Measurements at days 7 to 12 after onset of menstrual cycle. ^f^Serum samples in early follicular phase: days 1 to 5 after onset of menstrual cycle. ^g^Daily salivary samples throughout one entire menstrual cycle.

We observed an association between two SNPs (*rs749292*, *rs7172156*) and both salivary estradiol and absolute mammographic density. Moreover, a positive association was observed between *rs749292* and midmenstrual salivary 17β-estradiol (*P* = 0.026), and an inverse association between *rs7172156* and midmenstrual salivary 17β-estradiol (*P* = 0.002), after adjustment for age, BMI and parity (Table 2). We also observed a negative association between *rs749292* and absolute mammographic density (*P* = 0.032) after adjusting for age, BMI, parity, salivary midmenstrual 17β-estradiol and serum HDL-C.

**Table 2:** **Associations between two** ***CYP19A1*** **single-nucleotide polymorphisms (** ***rs749292*** **,** ***rs7172156*** **) and 17β**-**estradiol**

***CYP19A1*** SNPs	Location	Frequencies	Genotype	β-value (95% CI)	***P*** -value
*rs749292*	Intron				
Salivary 17β-estradiol		0.283	*AA*	Reference	
		0.457	*Aa*	2.73 (−0.22, 5.68)	0.069
		0.26	*aa*	3.79 (0.39, 7.20)	0.029
*P*-value for trend					0.026
Serum 17β-estradiol			*AA*	Reference	
			*Aa*	6.77 (−13.4, 26.9)	0.509
			*aa*	0.73 (−22.8, 24.2)	0.951
*P*-value for trend					0.905
*rs7172156*	Intron				
Salivary 17β-estradiol		0.406	*AA*	Reference	
		0.444	*Aa*	−0.10 (−2.76, 2.56)	0.939
		0.15	*aa*	−6.96 (−10.6, −3.32)	<0.001
*P*-value for trend					0.002
Serum 17β-estradiol			*AA*	Reference	
			*Aa*	−3.38 (−22.1, 15.3)	0.722
			*aa*	*−*12.4 (−38.1, 13.2)	0.340
*P*-value for trend					0.365

Multivariable linear regression model adjusted for age, parity and body mass index. β: Estimated slope coefficient (for example, change in response) from reference (*AA*) to *Aa* and *aa*; CI, Confidence interval; SNP, Single-nucleotide polymorphism. Salivary midmenstrual estradiol is the average of aligned menstrual estradiol levels from days −7 to +6. Serum 17β-estradiol was measured from early follicular phase days 1 to 5.

### *rs749292, rs7172156*and oestrogen levels

The associations between *rs749292* and *rs7172156* with 17β-estradiol were studied further with multivariable linear regression analyses. For *rs749292*, we observed a positive association between the minor homozygous genotype (*aa*) and salivary 17β-estradiol (β = 3.79, *P* = 0.03). For *rs7172156*, we observed an inverse association between the minor homozygous genotype and salivary 17β-estradiol (β = −6.96, *P* < 0.001) (Table 2). We then dichotomized participants by median split of BMI (23.6 kg/m^2^). For *rs7172156*, the minor homozygous genotype (*aa*) was inversely associated with 17β-estradiol levels (*aa*: β = −10.2, *P* < 0.001) in women with a high BMI (>23.6 kg/m^2^) (Table 3).

**Table 3:** **Associations between the** ***CYP19A1*** **single-nucleotide polymorphisms (** ***rs749292*** **,** ***rs7172156*** **) and 17β**-**estradiol by median body mass index (23.6 kg/m** ^**2**^**)**

	Genotype	β-value (95% CI)	***P*** -value
*rs749292*			
Salivary 17β-estradiol			
BMI ≤23.6 kg/m^2^	*AA*	Reference	
	*Aa*	2.72 (−1.06, 6.50)	0.157
	*aa*	2.79 (−1.54, 7.12)	0.203
*P*-value for trend			0.197
BMI >23.6 kg/m^2^	*AA*	Reference	
	*Aa*	3.08 (−1.79, 7.96)	0.212
	*aa*	5.26 (−0.32, 10.8)	0.064
*P*-value for trend			0.059
*rs7172156*			
Salivary 17β-estradiol			
BMI ≤23.6 kg/m^2^	*AA*	Reference	
	*Aa*	0.78 (−2.63, 4.19)	0.650
	*aa*	−3.98 (−9.11, 1.14)	0.126
*P*-value for trend			0.326
BMI >23.6 kg/m^2^	*AA*	Reference	
	*Aa*	−1.26 (−5.62, 3.11)	0.569
	*aa*	−10.2 (−15.7, −4.68)	<0.001
*P*-value for trend			0.001

Multivariable linear regression model adjusted for age, body mass index (BMI) and parity. β: Estimated slope coefficient (for example, change in response) from reference value (*AA*) to *Aa* and *aa*. Salivary midmenstrual 17β-estradiol is the average of aligned menstrual estradiol levels from days −7 to +6.

**Figure 1:**
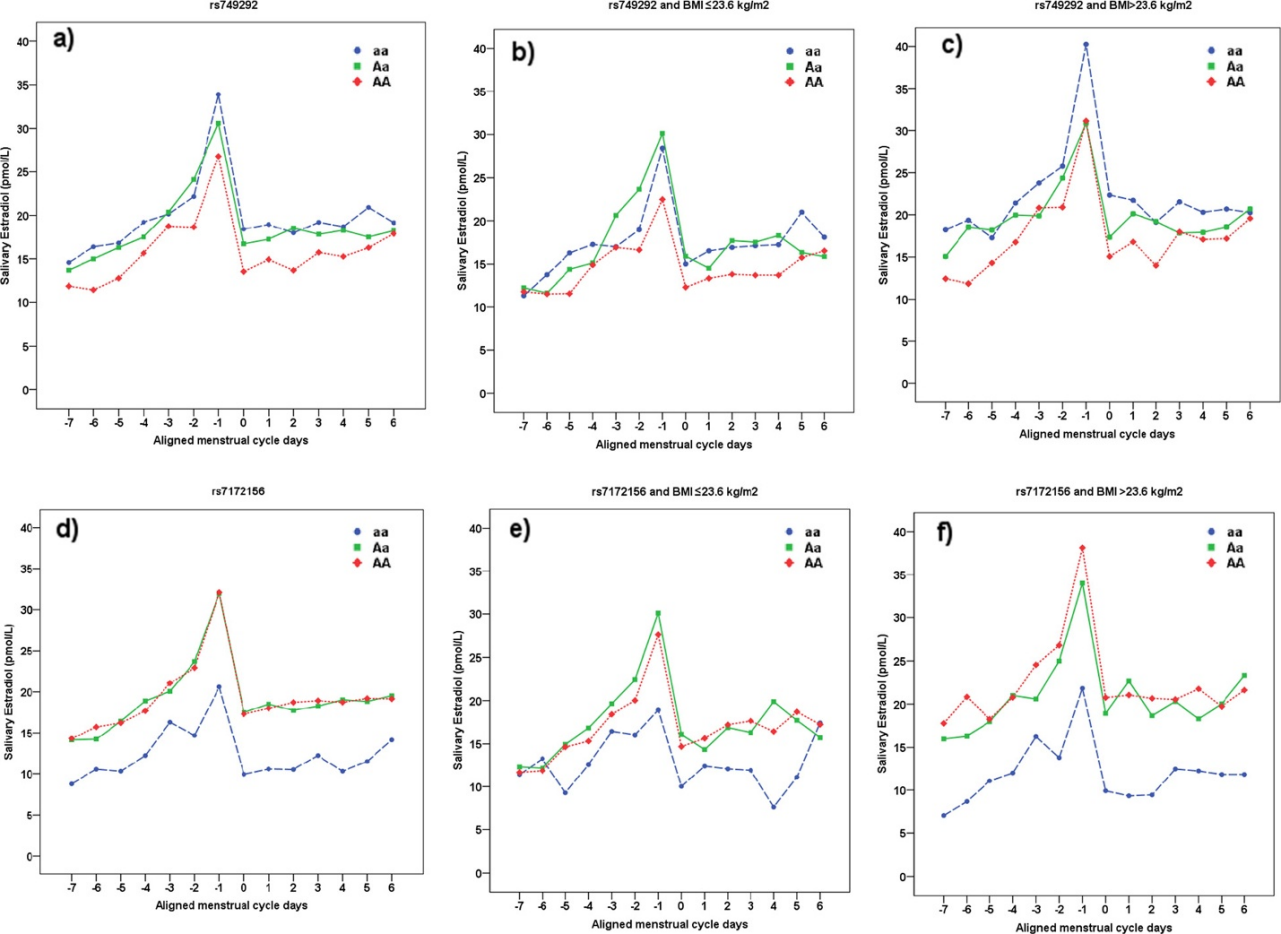
**Mean salivary 17β**-**estradiol levels across menstrual cycles for** ***rs749292*** **and** ***rs7172156*** **, adjusted for age, body mass index and parity. (a)** *rs749292* mean estradiol levels: minor homozygous genotype (*aa*) (*n* = 46), 19.8 pmol/L; heterozygous genotype (*Aa*) (*n* = 86), 18.7 pmol/L; and major homozygous genotype (*AA*) (*n* = 57), 16.0 pmol/L (*P* = 0.075). **(b)** *rs749292* and low body mass index (BMI ≤23.6 kg/m^2^): *aa* (*n* = 25), 17.5 pmol/L; *Aa* (*n* = 46), 17.4 pmol/L; and *AA* (*n* = 25), 14.7 pmol/L (*P* = 0.294). **(c)** *rs749292* and high BMI >23.6 kg/m^2^: *aa* (*n* = 21), 22.3 pmol/L; *Aa* (*n* = 40), 19.9 pmol/L; and *AA* (*n* = 30), 17.6 pmol/L (*P* = 0.265). **(d)** *rs7172156* mean estradiol levels: *aa* (*n* = 29), 12.3 pmol/L; *Aa* (*n* = 83), 19.2 pmol/L; and *AA* (*n* = 78), 19.3 pmol/L (*P* = 0.001). **(e)** *rs7172156* and low BMI ≤23.6 kg/m^2^: *aa* (*n* = 12), 12.9 pmol/L; *Aa* (*n* = 41), 17.5 pmol/L; and *AA* (*n* = 45), 16.9 pmol/L (*P* = 0.208). **(f)** *rs7172156* and high BMI >23.6 kg/m^2^: *aa* (*n* = 17), 12.0 pmol/L; *Aa* (*n* = 42), 20.9 pmol/L; and *AA* (n = 33), 22.4 pmol/L (*P* = 0.001).

No clear association was observed between any of these SNPs and serum levels of 17β-estradiol at any of the three measured time periods (early follicular, late follicular or late luteal phase) of the menstrual cycle. In the mixed linear regression models, we found that women with different genotypes of *rs7172156* varied in the levels of average midmenstrual salivary 17β-estradiol (*P* = 0.001). Among women with genotype *AA* and genotype *Aa*, compared with women with genotype *aa*, 57% and 56% higher mean 17β-estradiol levels were observed, respectively (Figure 1d). This association was even more marked when we dichotomised the data by median split of BMI (23.6 kg/m^2^). We observed an 87% lower level of mean 17β-estradiol throughout a menstrual cycle in heavier women (BMI >23.6 kg/m^2^) with minor genotype *aa* of *rs7172156* compared with those with major genotype *AA* (Figure 1f). Among women with genotype *AA*, heavier women had a 33% higher level of 17β-estradiol compared to leaner women. However, in genotype *aa*, there was no increase in 17β-estradiol levels when we compared leaner and heavier women. When comparing mean 17β-estradiol levels in lean women (BMI ≤23.6 kg/m^2^) with *rs749292* major genotype *AA* with heavier women (BMI >23.6 kg/m^2^) with *rs749292* minor genotype *aa*, a 52% higher mean 17β-estradiol level was observed (Figure 1).

### *rs749292* and *rs7172156*and mammographic density phenotypes

The association between the SNPs and mammographic density phenotypes was studied with multivariable linear regression models. For *rs749292*, we observed an inverse association between minor alleles (*Aa*, *aa*) and absolute mammographic density (Table 4). We observed a positive association between *rs7172156* minor genotype *aa* and absolute mammographic density.

**Table 4:** **Association between** ***CYP19A1*** **single-nucleotide polymorphisms (** ***rs749292*** **and** ***rs7172156*** **) and mammographic density phenotypes, overall and stratified by median body mass index (23.6 kg/m** ^**2**^ **)**

Mammographic density		Total		BMI ≤23.6		BMI >23.6	
	Genotype	β-value (95% CI)	***P*** -value	β-value (95% CI)	***P*** -value	β-value (95% CI)	***P*** -value
		( ***n*** = 202)		( ***n*** = 101)		( ***n*** = 101)	
*rs749292*							
Absolute density	*AA*	Reference		Reference		Reference	
	*Aa*	*−*7.78 (−15.5, −0.12)	0.047	−13.0 (−22.2, −3.82)	0.006	1.91 (−9.86, 13.7)	0.748
	*aa*	*−*9.47 (−18.3, −0.61)	0.036	−14.1 (−24.8,−3.44)	0.010	−1.36 (−14.9, 12.2)	0.842
*P*-value for trend			0.032		0.015		0.587
Percent density	*AA*	Reference		Reference		Reference	
	*Aa*	*−*2.64 (−7.68, 2.39)	0.301	−3.01 (−10.0, 4.01)	0.396	2.44 (−3.93, 8.82)	0.449
	*aa*	*−*2.68 (−8.50, 3.14)	0.364	−2.33 (−10.5, 5.81)	0.571	0.42 (−6.92, 7.76)	0.910
*P*-value for trend			0.348		0.537		0.866
*rs7172156*							
Absolute density	*AA*	Reference		Reference		Reference	
	*Aa*	0.27 (−6.70, 7.24)	0.939	3.94 (−4.63, 12.5)	0.363	−4.87 (−15.9, 6.15)	0.768
	*aa*	11.6 (1.43, 21.8)	0.026	18.2 (5.67, 30.8)	0.005	−2.15 (−16.6, 12.3)	0.382
*P*-value for trend			0.074		0.011		0.978
Percent density	*AA*	Reference		Reference		Reference	
	*Aa*	*−*1.52 (−6.09, 3.05)	0.512	1.15 (−5.38, 7.67)	0.728	−4.98 (−10.9, 0.93)	0.097
	*aa*	2.23 (−4.45, 8.91)	0.512	2.01 (−7.57, 11.6)	0.678	−2.26 (−9.99, 5.47)	0.563
*P*-value for trend			0.792		0.573		0.847

Multivariable linear regression model adjusted for age, parity, body mass index (BMI), 17-β-estradiol and high-density lipoprotein cholesterol. β: Estimated slope coefficient (for example, change in response) from reference (*AA*) to *Aa* and *aa*. Mammograms were taken within late follicular phase from days 7 to 12.

After dichotomising by median split of BMI, we found that *rs749292* minor alleles were inversely associated with absolute mammographic density (*Aa*: β = −13.0, *P* = 0.006; *aa*: β = −14.1, *P* = 0.010) in lean women (≤23.6 kg/m^2^), but not in women with a BMI >23.6 kg/m^2^. Among lean women (≤23.6 kg/m^2^) with *rs7172156* genotype *aa*, we observed a positive association with absolute mammographic density (*aa*: β = 18.2, *P* = 0.005) (Table 4).

In the multivariable logistic regression models, lean women (BMI ≤23.6 kg/m^2^) who had *rs749292* minor alleles (*Aa*, *aa*) had an 80% lower risk for high percent mammographic density (above median: >28.5%) (*Aa*: OR = 0.19 (95% CI, 0.05 to 0.82); *aa*: OR = 0.17 (95% CI 0.03 to 0.82)). The results were similar but attenuated for absolute mammographic density (Table 5).

**Table 5:** **Adjusted odds ratios for above-median absolute mammographic density (>32.4 cm** ^**2**^ **) and above-median percent mammographic density (>28.5%) by** ***CYP19A1*** **single-nucleotide polymorphism and stratified by median body mass index (23.6 kg/m** ^**2**^ **)**

Mammographic density	Genotype	Total (n = 202)	BMI ≤23.6 ( ***n*** = 101)	BMI >23.6 ( ***n*** = 101)
		OR (95% CI)	OR (95% CI)	OR (95% CI)
*rs749292*				
Absolute density	*AA*	1.0	1.0	1.0
	*Aa*	0.59 (0.29, 1.22)	0.23 (0.07, 0.75)	1.28 (0.45, 3.63)
	*aa*	0.86 (0.37, 1.98)	0.28 (0.08, 1.05)	2.21 (0.68, 7.15)
Percent density	*AA*	1.0	1.0	1.0
	*Aa*	0.57 (0.25, 1.30)	0.19 (0.05, 0.82)	1.41 (0.42,4.74)
	*aa*	0.64 (0.25, 1.64)	0.17 (0.03, 0.82)	1.85 (0.49,6.99)
*rs7172156*				
Absolute density	*AA*	1.0	1.0	1.0
	*Aa*	0.76 (0.39, 1.48)	1.49 (0.56, 3.97)	0.35 (0.13, 0.94)
	*aa*	1.16 (0.47, 2.88)	5.45 (1.13, 26.3)	0.34 (0.09, 1.25)
Percent density	*AA*	1.0	1.0	1.0
	*Aa*	0.85 (0.40, 1.82)	1.91 (0.64, 5.68)	0.40 (0.13, 1.22)
	*aa*	1.40 (0.51, 3.82)	5.48 (0.92, 32.7)	0.45 (0.11, 1.87)

Multivariable logistic regression adjusted for age, body mass index (BMI) and parity and stratified by median BMI (23.6 g/m^2^). Major homozygous genotype *AA*, heterozygous genotype *Aa* and minor homozygous genotype *aa*. Absolute mammographic density with median 32.4 cm^2^ as cutoff. Percent mammographic density with median 28.5% as cutoff. CI: Confidence interval; OR: Odds ratio.

For *rs7172156*, lean women with a minor homozygous genotype had a 5.45 higher OR for high absolute mammographic density (*aa*: OR = 5.45 (95% CI, 1.13 to 26.3)). Similar associations were observed for *rs7172156* and percent mammographic density (Table 5).

## Discussion

In the present study of premenopausal women, two SNPs (*rs749292*, *rs7172156*) of eight studied in the *CYP19A1* gene were related to both daily salivary 17β-estradiol and mammographic density phenotypes. The association with mammographic density was revealed when we used salivary 17β-estradiol as a covariate, and similar results were observed for absolute and percent mammographic density. Furthermore, our results suggest that body weight may modify these associations. We observed an 87% lower level of daily 17β-estradiol throughout a menstrual cycle in heavier women (BMI >23.6 kg/m^2^) with minor genotype *aa* (17β-estradiol 12.3 pmol/L) of *rs7172156* compared with major genotype *AA* (17β-estradiol 22.4 pmol/L)*.* Furthermore, lean women with *rs7172156* minor homozygous genotype *aa* had a fivefold higher OR for high absolute mammographic density compared with major homozygous genotype *AA*. Lean women who had *rs749292* minor alleles had 70% to 80% lower risk for high absolute and high percent mammographic density compared with major homozygous genotype *AA*.

The *CYP19* activity is responsible for the bioconversion of androgens to oestrogens [5],[6], and to our knowledge, there have been few studies related to *CYP19A1* SNPs, daily levels of oestrogen throughout an entire menstrual cycle and mammographic density phenotypes in premenopausal women. It is not clear why and how noncoding SNPs influence the gene activity, but previous genome-wide association studies have shown intronic SNPs to be important breast cancer risk loci [41]. This does not necessarily imply that the SNPs are causal, but it may help to identify novel susceptibility loci. In addition, intronic SNPs may regulate gene expression through endogenous *trans*-acting factors, epigenetics and chromosome conformation [42]. Our results are in part supported by a previous report [10] that *rs749292* minor alleles were associated with a 10% to 20% increase in oestrogen levels among postmenopausal women in a combined analysis of five cohort studies. Other SNPs in the *CYP19A1* gene have also been studied, and in one study on postmenopausal women with a mean age of 57 years and a mean BMI of 24.2 kg/m^2^, researchers found an association with circulating oestrogen levels, but only among women with BMI >25 kg/m^2^ [43]. Interestingly, functional genetic polymorphisms may also influence the level of estradiol in women undergoing inhibitory treatment, as two *CYP19A1* SNPs were associated with higher estradiol levels, particularly after initiation of aromatase inhibitors [44]. These findings imply that *CYP19A1* SNPs may be of clinical interest, as aromatase inhibitor treatment has been shown to be one of the most effective modern antihormonal breast cancer treatment regimens. To our knowledge, no clear associations have been observed between *CYP19A1* SNPs and mammographic density [45], and researchers in one study found no associations of oestrogen synthesis or oestrogen metabolism genes with mammographic density in a mixed population of perimenopausal, young postmenopausal and postmenopausal women [45]. Few known genetic variants predict both mammographic density and breast cancer risk, but Lindstrom *et al*. found an association between common variants in the *ZNF365* gene, which promotes genome stability under DNA damage, with both mammographic density and breast cancer development [20]. In addition, SNPs in the inflammatory gene interleukin-6 (*IL-6*) have recently been associated with premenopausal percent mammographic density [46]. Despite the clear association of endogenous oestrogens with breast cancer development [1], results have been inconsistent regarding associations between *CYP19A1* variants and risk for breast cancer [10],[47]-[49], but *rs1008805* [50] and, recently, *rs10046* were observed to be associated with breast cancer susceptibility among premenopausal women [51].

Elevated BMI has been related to higher levels of sex hormones in both premenopausal [30] and postmenopausal women [52], and weight loss through diet and exercise may reduce sex steroid hormone levels in premenopausal [53] and postmenopausal women [54]. We previously observed that *CYP17* polymorphisms were associated with 17β-estradiol levels, especially in women with unfavourable metabolic profiles [55]. Interestingly, in the present study, an inverse association was observed between *rs749292* minor alleles and absolute mammographic density among lean women, but this association disappeared in heavier women. Furthermore, *rs7172156* minor alleles were associated with higher absolute mammographic density among lean women. In contrast, we found that among women with minor alleles and high BMI, *rs7172156* may be a protective polymorphism associated with lower 17β-estradiol and lower OR for having above-median percent mammographic density (>28.5%) and absolute mammographic density (>32.4 cm^2^). Similar mammographic threshold estimates of 25% mammographic density and 32-cm^2^ absolute mammographic density have been shown to predict a two- to threefold risk of breast cancer development within 5 to 10 years [56],[57].

Interestingly, a previous study observed an association between *rs7172156*, *rs749292* [58] and serum levels of hepatocyte growth factor (HGF). HGF is a cytokine derived from adipose tissue [58] that promotes cell migration, proliferation and invasion, and previous studies have found associations between HGF levels and development from benign breast disorders to preinvasive, basal-like breast cancer [59], as well as further correlations with poor prognosis. These findings lead us to hypothesize that there may be a biological rational for the associations we observed for two SNPs in *CYP19A1*: *rs7172156* and *rs749292*.

Our study has several strengths. These are inclusion of premenopausal women; clinical measurements carefully timed to the menstrual cycle, including mammographic density phenotypes and serum and daily saliva 17β-estradiol; and a validated computer-assisted method for quantifying mammographic density. In contrast, we did not observe the same associations between these two SNPs in *CYP19A1* and serum 17β-estradiol as we did for salivary 17β-estradiol. Importantly, salivary 17β-estradiol was assessed daily, is the free bioavailable fraction and is not bound to albumin or sex hormone-binding globulin, in contrast to the serum 17β-estradiol levels, and may in part explain the variations observed [30],[33]. Previous research has indicated that single measurements of serum oestrogen do not accurately reflect women’s long-term oestrogen levels [3], whereas multiple measurements of unbound bioavailable levels probably give a picture of the real endogenous cumulative exposure over time. This means that single measurements are likely to be an underestimate because they do not capture the premenopausal cyclical changes and will be imperfect estimates of the true pattern [3],[60]. Thus, use of exploratory, noninvasive, repeated sampling of salivary hormones may provide new knowledge on the true association between hormones and breast cancer. Moreover, this may in part explain why circulating oestrogen levels consistently have been observed to increase risk, as well as risk prediction for invasive postmenopausal breast cancer [61], but the association between endogenous oestrogen levels and breast cancer among premenopausal women is less clear [3]. Today, liquid chromatography-tandem mass spectrometry, as compared to the immunoassay method, is a more efficient way of analysing salivary hormones with higher specificity and sensitivity. However, previous studies on estradiol measurements, specifically, have shown a correlation of 0.969 between mass spectrometry and immunoassays [62]. However, our sample size was small, and associations could have been missed by chance. Furthermore, the population was a sample of volunteer participants and therefore may not be representative of the source population, but their average BMI and other lifestyle-related factors and lipid profiles are in accordance with the population of premenopausal Norwegian women [63]. A limited number of SNPs were examined, based on the biological hypothesis that polymorphisms in the *CYP19* gene may influence 17β-estradiol levels and mammographic phenotypes. Even though only eight SNPs were examined, there is a risk of false-positive results. Nevertheless, our findings are intriguing and support future research in larger sample sizes.

## Conclusion

In the present study, we found associations between two *CYP19A1* SNPs (*rs7172156* and *rs749292*) and both daily 17β-estradiol throughout an entire menstrual cycle and both absolute and percent mammographic density in premenopausal women, and the results differed between lean and heavier women. This observation suggests that there may be genetic influences on these breast cancer biomarkers and also that the effect of body size may play a major role. Future research on genetic control of mammographic density phenotypes and sex hormones should include exploratory salivary hormone measurements and take body size and adiposity into account.

## Additional file

## Electronic supplementary material

Additional file 1: **Four supplementary tables. Table S1.** Allele frequencies and distributions of selected single-nucleotide polymorphisms (SNPs) in *CYP19A1*: The Norwegian EBBA-I study. **Table S2.** Population frequencies of single-nucleotide polymorphisms (SNPs) in selected single-nucleotide polymorphisms in *CYP19A1.* **Table S3.** Associations between each of eight selected single-nucleotide polymorphisms (SNPs) in the *CYP19A1* region and mammographic density (total breast area, absolute density, percent density and nondense breast areas). **Table S4.** Associations between each of eight selected single-nucleotide polymorphisms (SNPs) in the *CYP19A1* region and estradiol. (DOC 124 KB)

Below are the links to the authors’ original submitted files for images.

Authors’ original file for figure 1

## Abbreviations

BMI: Body mass index
CI: Confidence interval
CV: Coefficient of variation
EBBA-I: Norwegian Energy Balance and Breast cancer Aspects I study
ER: Oestrogen receptor
HGF: Hepatocyte growth factor
kb: Kilobase
HDL-C: High-density lipoprotein cholesterol
OR: Odds ratio
LDL-C: Low-density lipoprotein cholesterol
ROI: Region of interest
SNP: Single nucleotide polymorphism
UNN: University Hospital of North Norway, Tromsø
