# Supplementary material for: Gene variations in oestrogen pathways, CYP19A1, daily 17β-estradiol and mammographic density phenotypes in premenopausal women
Source: Breast Cancer Res. 2014 Dec 19;16:499. doi: 10.1186/s13058-014-0499-2 (PMC4303212; doi:10.1186/s13058-014-0499-2)
Supplement: Supplementary file 1 — Additional file 1: Four supplementary tables. Table S1. Allele frequencies and distributions of selected single-nucleotide polymorphisms (SNPs) in CYP19A1: The Norwegian EBBA-I study. Table S2. Population frequencies of single-nucleotide polymorphisms (SNPs) in selected single-nucleotide polymorphisms in CYP19A1. Table S3. Associations between each of eight selected single-nucleotide polymorphisms (SNPs) in the CYP19A1 region and mammographic density (total breast area, absolute density, percent density and nondense breast areas). Table S4. Associations between each of eight selected single-nucleotide polymorphisms (SNPs) in the CYP19A1 region and estradiol. (DOC 124 KB) [file 13058_2014_499_MOESM1_ESM.doc]

**Table S1 Allele frequencies and distributions of selected single nucleotide polymorphisms (SNPs) in *CYP19A1*: The Norwegian EBBA-I**

| **SNP** | **Location (base-pairs)** | **SNP position ^a^** | **Alleles** (HapMap alleles) | **MAF** | **HWE^b^** | ***AA*** | ***Aa*** | ***aa*** |
| --- | --- | --- | --- | --- | --- | --- | --- | --- |
|  |  |  |  |  |  |  |  |  |
| *rs10046* | 51502986 | 3UTR | C>T | 0.476 | 0.2495 | 0.302 | 0.442 | 0.257 |
| *rs17703883* | 51530097 | intron | C>T | 0.211 | 0.6171 | 0.632 | 0.316 | 0.053 |
| *rs2414097* | 51529835 | intron | A>G | 0.32 | 0.7506 | 0.47 | 0.421 | 0.109 |
| *rs2445761* | 51615616 | intron | C>T | 0.444 | 0.2661 | 0.333 | 0.439 | 0.227 |
| *rs4646* | 51502844 | 3UTR | A<C | 0.278 | 0.555 | 0.492 | 0.459 | 0.049 |
| *rs7172156* | 51546298 | intron | A<G | 0.372 | 0.6118 | 0.406 | 0.444 | 0.15 |
| *rs727479* | 51534547 | intron | G>T | 0.325 | 0.7485 | 0.462 | 0.425 | 0.113 |
| *rs749292* | 51558731 | intron | A<G | 0.487 | 0.388 | 0.283 | 0.457 | 0.26 |
|  |  |  |  |  |  |  |  |  |
| *A*, major allele; *a*, minor allele; HWE, Hardy–Weinberg equilibrium; MAF, minor allele frequency; SNP, single nucleotide polymorphism.  ^a^According to information on dbnSNP homepage: www.ncbi.nlm.nih.gov/projects/SNP/  ^b^HWE *p* value. | | | | | | | | |

**Table S2 Population frequencies of single nucleotide polymorphisms (SNPs) in selected single nucleotide polymorphisms in *CYP19A1***

| **SNP** | **Location** | **Switch** | **Genotype** | **EBBA-I** | **White European** | **Chinese** | **African** |
| --- | --- | --- | --- | --- | --- | --- | --- |
|  |  |  |  |  |  |  |  |
| *rs7172156* | Intron | A<G | *aa* | 0.15 |  |  |  |
|  |  |  | *Aa* | 0.444 |  |  |  |
|  |  |  | *AA* | 0.406 |  |  |  |
|  |  |  | MAF | 0.372 | 0.35 | 0.22 | 0.26 |
|  |  |  |  |  |  |  |  |
| *rs749292* | Intron | A<G | *aa* | 0.26 |  |  |  |
|  |  |  | *Aa* | 0.457 |  |  |  |
|  |  |  | *AA* | 0.283 |  |  |  |
|  |  |  | MAF | 0.487 | 0.45 | 0.36 | 0.48 |
|  |  |  |  |  |  |  |  |

*A*, major allele; *a*, minor allele; MAF, minor allele frequency; SNP, single nucleotide polymorphism.

^a^According to information on dbnSNP homepage: www.ncbi.nlm.nih.gov/projects/SNP/

**Table S3 Associations between each of eight selected single nucleotide polymorphisms (SNPs) in the *CYP19A1* region and mammographic density (total breast area, absolute density, percent density, and non-dense breast areas)**

| **SNP** | **Mammographic density** | **β value^a^** | **95% CI ^a^** | ***p value^a^*** | **β value^b^** | **95% CI ^b^** | ***p value^b^*** |
| --- | --- | --- | --- | --- | --- | --- | --- |
|  |  |  |  |  |  |  |  |
| *rs10046* | Total area | −0.27 | (−8.59, 8.06) | *0.950* | −2.02 | (−10.6, 6.56) | *0.643* |
|  | Absolute density | −0.77 | (−5.09, 3.55) | *0.725* | −1.71 | (−6.23, 2.81) | *0.457* |
|  | Percent density | −0.15 | (−2.93, 2.63) | *0.916* | −0.65 | (−3.59, 2.29) | *0.662* |
|  | Non-dense area | 0.51 | (−7.50, 8.51) | *0.901* | −0.31 | (−8.57, 7.95) | *0.941* |
| *rs17703883* | Total area | −5.90 | (−15.9, 4.11) | *0.247* | −8.28 | (−18.5, 1.95) | *0.112* |
|  | Absolute density | 0.20 | (−5.02, 5.43) | *0.939* | −0.78 | (−6.22, 4.67) | *0.779* |
|  | Percent density | 2.12 | (−1.22, 5.46) | *0.213* | 1.62 | (−1.90, 5.14) | *0.364* |
|  | Non-dense area | −6.10 | (−15.7, 3.52) | *0.213* | −7.50 | (−17.4, 2.35) | *0.135* |
| *rs2414097* | Total area | −1.33 | (−10.0, 7.38) | *0.764* | −6.47 | (−15.3, 2.36) | *0.150* |
|  | Absolute density | 0.95 | (−3.58, 5.48) | *0.678* | 0.07 | (−4.62, 4.76) | *0.976* |
|  | Percent density | 2.04 | (−0.86, 4.93) | *0.167* | 1.70 | (−1.32, 4.73) | *0.268* |
|  | Non−dense area | −2.28 | (−10.7, 6.09) | *0.591* | −6.54 | (−15.0, 1.95) | *0.130* |
| *rs2445761* | Total area | −2.10 | (−10.9, 6.67) | *0.638* | −4.61 | (−13.6, 4.37) | *0.312* |
|  | Absolute density | −0.68 | (−5.23, 3.87) | *0.769* | −2.21 | (−6.94, 2.53) | *0.359* |
|  | Percent density | −0.03 | (−2.95, 2.91) | *0.987* | −0.48 | (−3.56, 2.60) | *0.757* |
|  | Non-dense area | −1.42 | (−9.84, 7.01) | *0.741* | −2.40 | (−11.0, 6.24) | *0.584* |
| *rs4646* | Total area | −7.41 | (−17.2, 2.39) | *0.138* | −6.96 | (−17.3, 3.33) | *0.184* |
|  | Absolute density | 0.23 | (−4.90, 5.36) | *0.929* | 1.12 | (−4.35, 6.59) | *0.687* |
|  | Percent density | 1.01 | (−2.78, 4.30) | *0.546* | 1.68 | (−1.85, 5.22) | *0.348* |
|  | Non-dense area | −7.64 | (−17.1, 1.78) | *0.111* | −8.08 | (−18.0, 1.81) | *0.108* |
| *rs7172156* | Total area | 7.38 | (−1.19, 15.9) | *0.091* | 6.69 | (−2.38, 15.8) | *0.147* |
|  | Absolute density | 2.84 | (−1.63, 7.31) | *0.212* | 4.35 | (−0.43, 9.13) | *0.074* |
|  | Percent density | −0.47 | (−3.35, 2.41) | *0.749* | 0.42 | (−2.70, 3.54) | *0.792* |
|  | Non-dense area | 4.54 | (−3.73, 12.8) | *0.281* | 2.34 | (−6.43, 11.1) | *0.599* |
| *rs727479* | Total area | −1.77 | (−10.4, 6.90) | *0.688* | −6.84 | (−15.6, 1.94) | *0.126* |
|  | Absolute density | 0.96 | (−3.55, 5.47) | *0.675* | 0.52 | (−4.62, 4.72) | *0.983* |
|  | Percent density | 2.16 | (−0.73, 5.03) | *0.142* | 1.81 | (−1.21, 4.82) | *0.238* |
|  | Non-dense area | −2.73 | (−11.1, 5.60) | *0.519* | −6.89 | (−15.3, 1.55) | *0.109* |
| ***rs749292*** | **Total area** | −6.98 | (−15.1, 1.17) | *0.093* | **−9.66** | **(−18.0, 1.28)** | ***0.024*** |
|  | **Absolute density** | −3.90 | (−8.12, 0.32) | *0.070* | **−4.83** | **(−9.25, −0.41)** | ***0.032*** |
|  | Percent density | −0.92 | (−3.66, 1.82) | *0.508* | −1.38 | (−4.28, 1.52) | *0.348* |
|  | Non-dense area | −3.08 | (−10.9, 4.80) | *0.442* | −4.82 | (−13.0, 3.32) | *0.244* |
|  |  |  |  |  |  |  |  |
| ^a^ Multivariable linear regression, adjusted by age, BMI, and parity.  ^b^ Multivariable linear regression, adjusted by age, BMI, parity, high-density lipoprotein-cholesterol, and mean mid-menstrual salivary 17β-estradiol, cycle days −7 to +6. | | | | | | | |

**Table S4 Associations between each of eight selected single nucleotide polymorphisms (SNPs) in the *CYP19A1* region and estradiol**

| **SNP** | **Hormone** | **β value** | **95% CI** | ***p value*** |
| --- | --- | --- | --- | --- |
| *rs10046* | Salivary mid-menstrual estradiol | 1.36 | (−0.38, 3.10) | *0.125* |
|  | Serum follicular estradiol | 0.01 | (−0.01, 0.02) | *0.437* |
| *rs17703883* | Salivary mid-menstrual estradiol | 1.25 | (−0.87, 3.37) | *0.245* |
|  | Serum follicular estradiol | 0.01 | (−0.01, 0.02) | *0.288* |
| *rs2414097* | Salivary mid-menstrual estradiol | 0.79 | (−1.03, 2.61) | *0.391* |
|  | Serum follicular estradiol | 0.00 | (−0.01, 0.02) | *0.561* |
| *rs2445761* | Salivary mid-menstrual estradiol | 0.02 | (−1.84, 1.87) | *0.987* |
|  | Serum follicular estradiol | −0.01 | (−0.02, 0.00) | *0.137* |
| *rs4646* | Salivary mid-menstrual estradiol | −1.25 | (−3.32, 0.83) | *0.238* |
|  | Serum follicular estradiol | −0.01 | (−0.02, 0.01) | *0.501* |
| ***rs7172156*** | **Salivary mid-menstrual estradiol** | **−2.74** | **(−4.49, −0.98)** | ***0.002*** |
|  | Serum follicular estradiol | −0.01 | (−0.02, 0.01) | *0.365* |
| *rs727479* | Salivary mid-menstrual estradiol | 0.70 | (−1.12, 2.51) | *0.451* |
|  | Serum follicular estradiol | 0.01 | (−0.01, 0.02) | *0.555* |
| ***rs749292*** | **Salivary mid-menstrual estradiol** | **1.94** | **(0.24, 3.63)** | ***0.026*** |
|  | Serum follicular estradiol | 0.00 | (−0.01, 0.01) | *0.905* |
|  |  |  |  |  |
| Multivariable linear regression, adjusted for age, BMI, and parity.  Salivary mid-menstrual estradiol: average levels across menstrual cycle days −7 to +6.  Serum estradiol taken days 1–5. | | | | |
