# Supplementary figures and images for: Gene variations in oestrogen pathways, CYP19A1, daily 17β-estradiol and mammographic density phenotypes in premenopausal women
Source: Breast Cancer Res. 2014 Dec 19;16:499. doi: 10.1186/s13058-014-0499-2 (PMC4303212; doi:10.1186/s13058-014-0499-2)

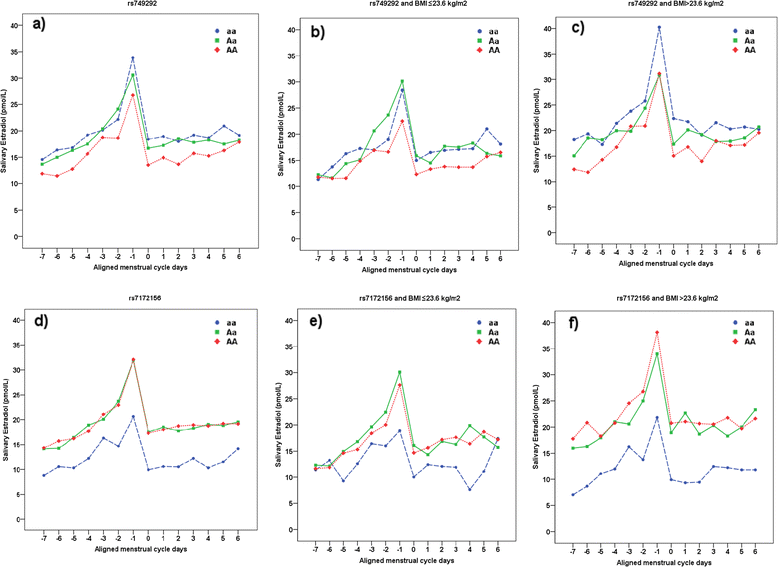

Supplement: Supplementary file 2 — Authors’ original file for figure 1 [file 13058_2014_499_MOESM2_ESM.gif]
